# The complete mitochondrial DNA sequence of the pantropical earthworm *Pontoscolex corethrurus* (Rhinodrilidae, Clitellata): Mitogenome characterization and phylogenetic positioning

**DOI:** 10.3897/zookeys.688.13721

**Published:** 2017-08-08

**Authors:** Ana C. Conrado, Hugo Arruda, David W.G. Stanton, Samuel W. James, Peter Kille, George Brown, Elodie Silva, Lise Dupont, Shabnam Taheri, Andrew J. Morgan, Nelson Simões, Armindo Rodrigues, Rafael Montiel, Luis Cunha

**Affiliations:** 1 Universidade Federal do Paraná, Departamento de Ciências do Solo, Curitiba, Paraná, Brazil; 2 CIRN/Departamento de Biologia, Universidade dos Açores, Ponta Delgada, Portugal; 3 Cardiff School of Biosciences, Cardiff University, Museum Avenue, Cardiff, UK CF10 3AX; 4 Maharishi University of Management, Sustainable Living Dept., Fairfield,IA, USA; 5 EMBRAPA-Florestas, Empresa Brasileira de Pesquisa Agropecuária (Embrapa), Colombo, PR, Brazil; 6 Université Paris Est Créteil, Institut d’écologie et des sciences de l’environnement de Paris (IEES), Créteil, France; 7 Centro de Vulcanologia e Avaliação de Riscos Geológicos (CVARG), Universidade dos Açores, Ponta Delgada, Portugal; 8 Laboratorio Nacional de Genómica para la Biodiversidad, Unidad de Genómica Avanzada, Centro de Investigación y de Estudios Avanzados del Instituto Politécnico Nacional, Irapuato, México

**Keywords:** Pontoscolex corethrurus, mitochondria, mitochondrial genome, Rhinodrilidae, earthworm, Azores, peregrine species

## Abstract

*Pontoscolex corethrurus* (Müller, 1857) plays an important role in tropical soil ecosystems and has been widely used as an animal model for a large variety of ecological studies in particular due to its common presence and generally high abundance in human-disturbed tropical soils. In this study we describe the complete mitochondrial genome of the peregrine earthworm *P. corethrurus*. This is the first record of a mitochondrial genome within the Rhinodrilidae family. Its mitochondrial genome is 14 835 bp in length containing 37 genes (13 protein-coding genes (PCG) 2 rRNA genes and 22 tRNA genes). It has the same gene content and structure as in other sequenced earthworms but unusual among invertebrates it hasseveral overlapping open reading frames. All genes are encoded on the same strand. Most of the PCGs use ATG as the start codon except for ND3 which uses GTG as the start codon. The A+T content of the mitochondrial genome is 59.9% (31.8% A 28.1% T 14.6% G and 25.6% for C). The annotated genome sequence has been deposited in GenBank under the accession number KT988053.

## Introduction

Excluding a few aquatic taxa, earthworms (Annelida: Clitellata) are mostly terrestrial and include ca. 5,500 species (Blakemore et al. 2006). Believed to have originated in the Guyana Shield (Righi 1984), the earthworm *Pontoscolex corethrurus* (Müller, 1857) is a globally distributed species found in most tropical regions. It mainly occurs in human-disturbed areas and can be used as an indicator of ecosystem disturbance (Brown et al. 2006), and is commonly used in ecotoxicological studies (e.g. Buch et al. 2011; Buch et al. 2013; Da Silva et al. 2016). The species formerly belonged in the Glossoscolecidae family, but was recently allocated to the Rhinodrilidae family by James (2012), following the phylogeny of James and Davidson (2012). It is also the most well-known earthworm species in the humid tropics, frequently used in ecological and agronomic studies (Bhattacharjee and Chaudhuri 2002; Buch et al. 2013; Chapuis-Lardy et al. 2010; Dupont et al. 2012; Hamoui 1991; Marichal et al. 2010). Being a geophagous endogeic species, *P. corethrurus* shows high plasticity regarding its tolerance to soil physicochemical characteristics, including variable moisture, high temperatures, exceptionally high carbon dioxide and low oxygen levels, and is capable of inhabiting nutrient-poor soils (Cunha et al. 2014; Hamoui 1991; Lavelle et al. 1987), as well as rotten logs (Buch et al. 2011).

Molecular data have become increasingly important in recent years. In animals, the mitochondrial DNA (mtDNA) typically contains 37 genes, encoding 13 proteins for the enzymes required for oxidative phosphorylation, the two ribosomal RNA units (rRNA), and 22 transfer RNAs (tRNAs) necessary for the translation of the proteins encoded by mtDNA (Anderson et al. 1981; Boore 1999; Zhao et al. 2015). Remarkable progress has been made over the past several years in the field of the molecular systematics of annelids. Compared with individual genes, the mitochondrial genome is still a promising tool for inferring phylogenetic relationships due to its high content of information, and has been applied in some phylogenetic studies involving earthworms (Zhang et al. 2015; Zhang et al. 2016b).

In this study, we sequenced the complete mtDNA sequence of *P. corethrurus* for the first time and analyzed its structure. Additionally, we conducted phylogenetic analyses based on the mitochondrial sequence data available elsewhere with the purpose of investigating the phylogenetic position of *P. corethrurus* within Clitellata. The information reported in this article will facilitate further investigations of phylogenetic relationships of different Annelida species.

## Material and methods

### Sample collection and DNA extraction

A group of clitellate (adult) *P. corethrurus* was collected in São Miguel Island (Azores, Portugal) inside pineapple greenhouses (Locality: Fajã de Baixo, 37°45'12.2"N, 25°38'21.3"W) during January 2015. Animals were euthanized in 10% ethanol and preserved in 96% ethanol for later work. A piece of body wall tissue was used for genomic DNA extraction using standard phenol/chloroform (Sambrook and Russell 2001) procedure followed by ethanol precipitation and kept at 4°C for subsequent use.

### Mitochondrial DNA amplification

The complete *P. corethrurus* mitogenome was amplified using seven sets of primers (Table 1) designed based on sequences retrieved from a previous study (Cunha et al. 2014).

**Table 1.** Details of the primers used to amplify the mitochondrial DNA of *P. corethrurus*.

Primer code	Orientation	Annealing position (bp)	Nucleotide sequence (5’-3’)	Melting Temperature (°C)
FP_1	Forward	2154..2175	CTCTACTATGTACCCAGGAGTG	57.46
RP_1	Reverse	2758..2775	GCGGCCAAGATAAAGCAC	57.67
RP_2	Reverse	3740..3762	TAGAGGCGGTAAGGAGAAAGTAT	58.61
RP_3	Reverse	5691..5708	CAGAGGCGAGGTAAATTC	53.85
RP_4	Reverse	6356..6373	TGTTCAGGGCTAGGATTG	54.99
FP_5	Forward	7983..8004	ACTAGTGTCACTTACAACAACC	57.16
RP_5	Reverse	8649..8670	TGATAAGGGGGAAAGTCTGATC	56.84
FP_6	Forward	8766..8787	AGTAGCCGCTATAATAGTCCTT	57.91
RP_6	Reverse	10328..10349	TGATTTGGGGTCAGAGCCGTAG	61.59
FP_7	Forward	10459..10478	AAAGCTTGGCGGTGCTTCAC	63.23
RP_7	Reverse	11242..11263	CCTAGTGTGTGTCAGGACGCTT	64.75

Long PCR targets were amplified using different combinations of the primer sets, and initially sequenced with the same forward or reverse primers. Subsequent primer walking method was used to close the sequencing gaps. To ensure the accuracy of the sequence, every two contiguous segments overlapped by at least 80 bp. PCRs were performed using ~40 ng of DNA and 0.4 μM forward and reverse primers, 0.2 mM dNTP mix and 1.25 U Platinum HiFi DNA polymerase buffered with 1X Mg-free buffer (Thermofisher Scientific, UK). PCR amplification buffer was supplemented with MgCl_2_ to achieve a final concentration of 1.75 mM in a total volume of 25 μl reaction mixture. The reaction was denatured at 95°C for 3 min, cycled 35 times at 95°C for 30 s, 30 s at the required primer annealing temperature and 72°C for 1 min per 1000 bp depending on target fragment length. Negative controls were included in all PCR amplifications to confirm the absence of contaminants. Before sequencing, PCR cleanups were performed using Exo-SAP-IT (Amersham Pharmacia, UK) reagents. Exonuclease 1 (0.25 μl) and Shrimp Alkaline Phosphatase (0.5 μl) were mixed with the PCR product (10 μl) and incubated at 37°C for 45 min followed by 80°C for 15 min. DNA was sequenced using ABI PRISM^®^ BigDye v3.1 Terminator Sequencing technology (Applied Biosystems, USA) on an ABI PRISM^®^ 3100 DNA automated Sequencer.

### Sequence editing and analysis

Sequence trace files were corrected and aligned with the MEGA v.7 (Kumar et al. 2016). The sequence overlap and mitogenome assembly were performed using CLC main workbench v.6 (Qiagen). The annotation of the 13 protein-coding genes and two rRNA genes were determined using the MITOS v.2 web server (Bernt et al. 2013) and manually curated using other published annelid mitogenomes as shown in Table 2, whereas the tRNA genes were identified using the program tRNAscan-SE 1.21 (Lowe and Eddy 1997). The annotated genome sequence was deposited in GenBank under accession number KT988053.

### Phylogenetic analyses

To clarify the phylogenetic position of *P. corethrurus* within the Clitellata, the complete mitogenome sequences of eight representative Clitellata species (Table 2) were incorporated together with the presently obtained *P. corethrurus* mitogenome sequence for phylogenetic analysis. Phylogenetic analyses were based on 13 protein-coding genes and the two rRNA units, which were aligned separately using MEGA v.7 (Kumar et al. 2016) with minor manual adjustments and then concatenated. The possible bias of substitution saturation at each codon position of protein-coding genes and two rRNA genes was investigated using DAMBE v.4.5.57 (Xia and Xie 2001) and MEGA v. 7 (Kumar et al. 2016).

**Table 2.** Representative Clitellata species included in this study for comparison.

**Species**	**Family**	**Length (bp)**	**GenBank accession number**
*Pontoscolex corethrurus*	Rhinodrilidae	14,835	**Present study**
*Tonoscolex birmanicus*	Megascolecidae	15,170	KF425518
*Amynthas gracilis*	Megascolecidae	15,161	NC_027258
*Duplodicodrilus schmardae*	Megascolecidae	15,156	NC_029867
*Metaphire guillemi*	Megascolecidae	15,174	NC_029869
*Perionyx excavatus*	Megascolecidae	15,083	NC_009631
*Lumbricus terrestris*	Lumbricidae	14,998	NC_001673
*Drawida japonica*	Moniligastridae	14,648	NC_028050
*Hirudo nipponia*	Hirudinidae	14,414	NC_023776

Two different methods, Bayesian inference (BI) and maximum likelihood (ML) were used to construct the phylogenetic tree. Bayesian analyses were undertaken with MrBayes v.3.1.2 (Ronquist and Huelsenbeck 2003) under the best-fit model of nucleotide evolution selected in MrModeltest v.2.3 (Nylander 2004), using the Assignment Index Criterion (AIC). Analyses were run for 1,000,000 generations, and sampled every 100 generations to assess convergence. Trees that produced non-stationary log-likelihood values were discarded as part of a burn-in procedure and combined the remaining trees that resulted in convergent log-likelihood scores from both independent searches. These trees were used to construct a consensus tree.

Maximum likelihood analysis (ML) was performed with MEGA v.7 (Kumar et al. 2016). Initial tree(s) for the heuristic search were obtained automatically by applying Neighbour-Joining and BioNJ algorithms to a matrix of pairwise distances estimated using the Maximum Composite Likelihood (MCL) approach and then selecting the topology with superior log likelihood value. A discrete Gamma distribution was used to model evolutionary rate differences among sites (5 categories (+G, alpha parameter = 0.9143)). The rate variation model allowed for some sites to be evolutionarily invariable ([+I], 17.7596% sites). The analysis involved nine mitogenome sequences (Table 2). All positions containing gaps and missing data were eliminated. The bootstrap consensus tree inferred from 1000 replicates was taken to represent the evolutionary history of the taxa analysed (Felsenstein 1985).

## Results and discussion

### Mitochondrial genomic structure

**Figure 1.**
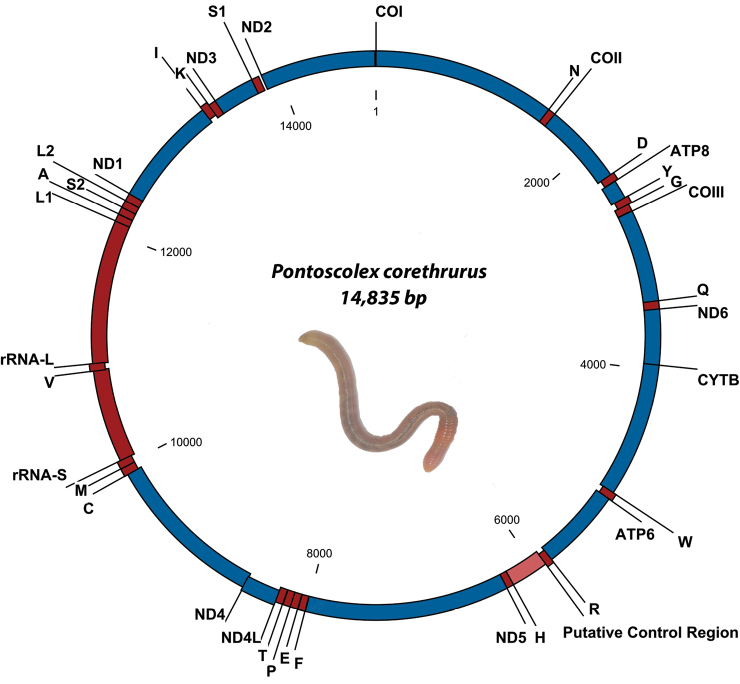
The mitochondrial genome of *Pontoscolex corethrurus* (Müller, 1857). Gene order and positions are shown, including the putative control region. IUPAC single letter codes are used to identify transfer RNA. The L1, L2, S1, and S2 transfer RNAs are differentiated on the basis of their anti-codons TAG, TAA, TCT, and TGA, respectively.

The mitochondrial genome of *P. corethrurus* was determined to be 14 835 bp in length, comprising 13 protein-coding genes (PCGs), 22 transfer RNAs (tRNAs), two ribosomal RNAs (rRNAs), and one putative control region with a length of 318 bp (Figure 1).

The mitochondrial genome structure is detailed in Table 3. Gene order and orientation are similar to the previous earthworm mitochondrial genomes (Boore and Brown 1995; Zhang et al. 2015) but slightly smaller and more condensed (with several intergenic overlaps, see Table 3). The gene organization is similar to other earthworm species (e.g. *Lumbricus terrestris*: Boore and Brown 1995).

**Table 3.** Organisation and structure of the *P. corethrurus* mitochondrial genome.

Gene	Direction	From	To	Size (bp)	Start	Stop	Anti-codon	Intergenic bases (bp)
COX1	+	1	1540	1540	ATG	T--		0
tRNA-^Asn^	+	1541	1602	62			GTT	0
COX2	+	1603	2289	687	ATG	TAG		-1
tRNA-^Asp^	+	2289	2351	63			GTC	2
ATP8	+	2354	2513	160	ATG	T--		0
tRNA-^Tyr^	+	2514	2576	63			GTA	-1
tRNA-^Gly^	+	2576	2638	63			TCC	3
COX3	+	2642	3419	778	ATG	T--		0
tRNA-^Gln^	+	3420	3488	69			TTG	0
ND6	+	3489	3954	466	ATG	T--		0
Cytb	+	3955	5094	1140	ATG	TAA		-2
tRNA-^Trp^	+	5092	5154	63			TCA	1
ATP6	+	5156	5851	696	ATG	TAA		-2
tRNA-^Arg^	+	5850	5910	61			TCG	0
Putative Control Region	+	5911	6228	318				
tRNA-^His^	+	6229	6288	60			GTG	0
ND5	+	6289	8010	1722	ATG	TAA		3
tRNA-^Phe^	+	8014	8073	60			GAA	4
tRNA-^Glu^	+	8078	8141	64			TTC	0
tRNA-^Pro^	+	8142	8204	63			TGG	4
tRNA-^Thr^	+	8209	8272	64			TGT	0
ND4L	+	8273	8569	297	ATG	TAA		-7
ND4	+	8563	9921	1359	ATG	TAA		-2
tRNA-^Cys^	+	9920	9986	67			GCA	1
tRNA-^Met^	+	9988	10050	63			CAT	-1
s-rRNA	+	10050	10838	789				-7
tRNA-^Val^	+	10832	10894	63			TAC	-2
l-rRNA	+	10893	12104	1212				0
tRNA-^Leu 1^	+	12105	12166	62			TAG	2
tRNA-^Ala^	+	12169	12230	62			TGC	1
tRNA-^Ser 2^	+	12232	12293	62			TGA	1
tRNA-^Leu 2^	+	12295	12360	66			TAA	0
ND1	+	12361	13290	930	ATG	TAG		-1
tRNA-^lle^	+	13290	13354	65			GAT	0
tRNA-^Lys^	+	13355	13417	63			TTT	0
ND3	+	13418	13770	353	GTG	TA-		-1
tRNA-^Ser 1^	+	13770	13832	63			TCT	0
ND2	+	13833	14835	1003	ATG	T--		0

The nucleotide composition is asymmetric (31.9% A, 27.9% T, 14.9% G, and 25.3% for C) with an overall A+T content of 59.9%. One remarkable trait of metazoan mitogenomes is the strand-specific bias in nucleotide composition (Hassanin et al. 2005; Reyes et al. 1998). Such bias is measured as G/C-skew (G%-C%)/(G%+C%) and A/T-skew (A%-T%)/(A%+T%), respectively (Perna and Kocher 1995). The overall GC- and AT-skews of the H-strand of *P. corethrurus* mitogenome were -0.258 and 0.066, respectively, indicating a compositional bias associated with an excess of C over G nucleotides and a slight excess of A over T nucleotides on the H-strand.

### Protein-coding genes

The *P. corethrurus* genome contained the expected 13 protein-coding genes with a total of 11,131 bp in size, accounting for 75.03% of the whole mitogenome. Most of the PCGs are initiated with ATG codons, except for ND3 gene, which uses GTG as the initiation codon. Six PCGs (COX1, ATP8, COX3, ND6, ND3, and ND2) are terminated with an incomplete codon T or TA, which could be completed to TAA by polyadenylation post-transcriptionally (Ojala et al. 1981). COX2 and ND1 use TAG as a termination codon.

Nucleotide composition and codon usage frequencies were calculated from a concatenated sequence of all protein-coding genes on the H-strand. The base composition of protein-coding genes revealed a negative bias for A (14.4%), especially at second codon positions (12.9%, Table 4). For all protein genes, T was the most frequent nucleotide at the first and third positions whereas G was most frequent at the second position.

**Table 4.** Base composition for protein-coding, tRNA, and rRNA genes of *P. corethrurus* mitogenome.

Gene/Region	Base composition (%)				A+T (%)	Size (bp)
	T	C	A	G		
COX1	27.4	26.7	27.9	18.1	55.3	1,540
COX2	25.8	24.3	34.9	15.0	60.7	687
ATP8	24.4	26.9	36.9	11.9	61.3	160
COX3	27.3	27.5	25.7	19.5	53.0	778
ND6	28.3	26.0	30.3	15.5	58.6	466
Cytb	28.0	27.1	29.7	15.3	57.6	1,140
ATP6	29.6	29.2	30.6	10.6	60.2	696
ND5	27.7	26.7	31.7	13.9	59.4	1,722
ND4L	25.9	28.3	33.0	12.8	58.9	297
ND4	27.5	27.5	32.3	12.7	59.8	1,359
ND1	28.4	25.8	29.8	16.0	58.2	930
ND3	32.6	26.1	27.2	14.2	59.8	353
ND2	30.0	28.2	30.7	11.1	60.7	1,003
Protein Coding						
1st	30.2	25.4	17.4	27.0	47.6	3,710
2st	24.9	27.6	12.9	34.6	37.8	3,710
3st	36.1	27.9	13.8	22.3	49.8	3,710
Total	30.4	27.0	14.7	28.0	45.1	11,131
tRNA	30.5	17.6	34.9	17.0	65.5	1,391
rRNA	24.1	22.0	37.6	16.3	61.7	2,001
Putative Control Region	31.5	18.9	36.2	13.5	67.6	318
Overall	28.1	25.6	31.8	14.6	59.9	14,835

### Ribosomal and transfer RNA genes

Like other mitochondrial genomes (Inoue et al. 2000; Zardoya et al. 1995), twenty-two tRNA genes were identified (Supplementary figure 1). The tRNA genes were scattered throughout the mitochondrial genome and ranged in size from 60 to 67 bp (Table 3). The *P. corethrurus* mitogenome also contained a small subunit of rRNA and a large subunit of rRNA, which were 789 bp and 1212 bp in length, respectively. As in other Clitellata genomes, these genes were located between the tRNA^Met^ and tRNA^Val^ genes and between tRNA^Val^ and tRNA^Leu^ genes, respectively (Zhang et al. 2016b).

### Non-coding regions

As shown in Table 3, there are 22 intergenic spacer regions, ranging in size from -7 to 4 bp observed in *P. corethrurus*.

As in most Clitellata, the major non-coding region in *P. corethrurus* mitochondrial genome was located between tRNA-^Arg^ and tRNA-^His^. It was determined to be 318 bp in length, less than other reported Clitellata species (Zhang et al. 2016a), and it had a base composition that was rich in A and T (A+T=67.6%).

### Phylogenetic analyses within the Clitellata

**Figure 2.**
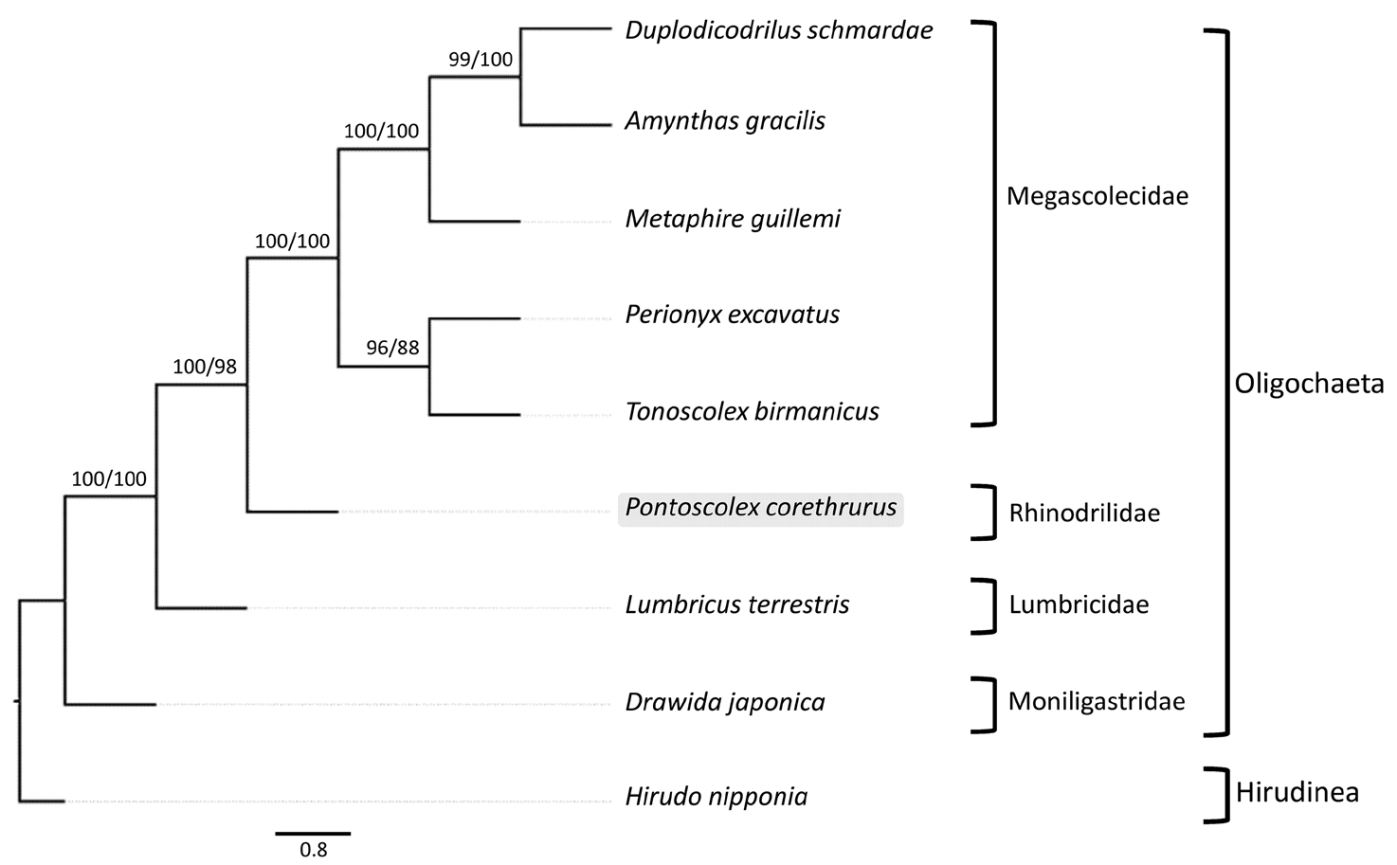
Phylogenetic relationships among phylum Annelida based on the combined 13,416 bp nucleotide positions. Total alignment length is greater than the combined *P. corethrurus* protein coding and rRNA sequence lengths due to overlapping protein coding sequences that are subsequently concatenated, and indel regions in the alignment. The posterior probability value of BI analyses and bootstrap support values of ML analyses (in the order: BI, ML) are indicated near the branches.

The phylogenetic trees (the 50% majority-rule consensus tree is shown in Figure 2) were highly consistent regardless of the analytic method used, and were statistically supported by high posterior probability and intermediate bootstrap values. This phylogenetic analysis represented the first investigation of *P. corethrurus* relationships within the Clitellata based on the complete mitogenome. As indicated by the tree, different species from the same family clustered together (Megascolecidae: *M. guillemi*, *D. schmardae*, *A. gracilis*, *P. excavatus* and *T. birmanicus*), and the species from Lumbricidae and the *P. corethrurus* formed a monophyletic group. The species *D. japonica* belongs to the Moniligastridae, the sister group to Crassiclitellata (earthworms), which explains its phylogenetic position. The Moniligastridae are not Crassiclitellata because they have a single cell layer in the clitellum.

## Conclusion

For the first time, the sequencing, annotation and analysis of the mitochondrial genome of a member of Rhinodrilidae was completed. The mitogenome of *P. corethrurus* was found to be 14,835 bp in length and showed a similar composition in size, low GC content and gene order to earthworm mitogenomes already available. The complete mitogenome reported here is expected to allow for further studies of the *P. corethrurus* phylogeny and for analyses on the taxonomic status of the family Rhinodrilidae.

## Declaration of interest section

The authors report no conflicts of interest and are responsible for the content and writing of the paper.

## Acknowledgments

We thank Jose Talavera for the taxonomic identification of the studied specimen. This study was financially supported by the Portuguese Government (FCT project PTDC/AAC-AMB/ 115713/2009). Luis Cunha was supported by an EU Marie Curie fellowship - MSCA-IF-2014-GF-660378. George Brown, Elodie da Silva acknowledge fellowship support by CNPq. Ana Caroline Conrado was supported by a scholarship funded by CAPES. Sam James was supported by USA NSF award DEB-1136604. Dave Stanton was supported by a NERC grant (NE/M017656/1).

## Supplementary materials

Supplementary material 1 Inferred secondary structure of 22 tRNA genes in the mitochondrial DNA of the pantropical earthworm *Pontoscolex corethrurus* (Rhinodrilidae, Clitellata). Data type: molecular data This dataset is made available under the Open Database License (http://opendatacommons.org/licenses/odbl/1.0/). The Open Database License (ODbL) is a license agreement intended to allow users to freely share, modify, and use this Dataset while maintaining this same freedom for others, provided that the original source and author(s) are credited.Ana C. Conrado, Hugo Arruda, David W.G. Stanton, Samuel W. James, Peter Kille, George Brown, Elodie Silva, Lise Dupont, Shabnam Taheri, Andrew J. Morgan, Nelson Simões, Armindo Rodrigues, Rafael Montiel8, Luis Cunha
