# Supplementary material for: The complete mitochondrial DNA sequence of the pantropical earthworm Pontoscolex corethrurus (Rhinodrilidae, Clitellata): Mitogenome characterization and phylogenetic positioning
Source: Zookeys. 2017 Aug 8;(688):1–13. doi: 10.3897/zookeys.688.13721 (PMC5672579; doi:10.3897/zookeys.688.13721)
Supplement: Supplementary material 1 — Inferred secondary structure of 22 tRNA genes in the mitochondrial DNA of the pantropical earthworm Pontoscolex corethrurus (Rhinodrilidae, Clitellata). [file zookeys-688-001-s001.pdf]

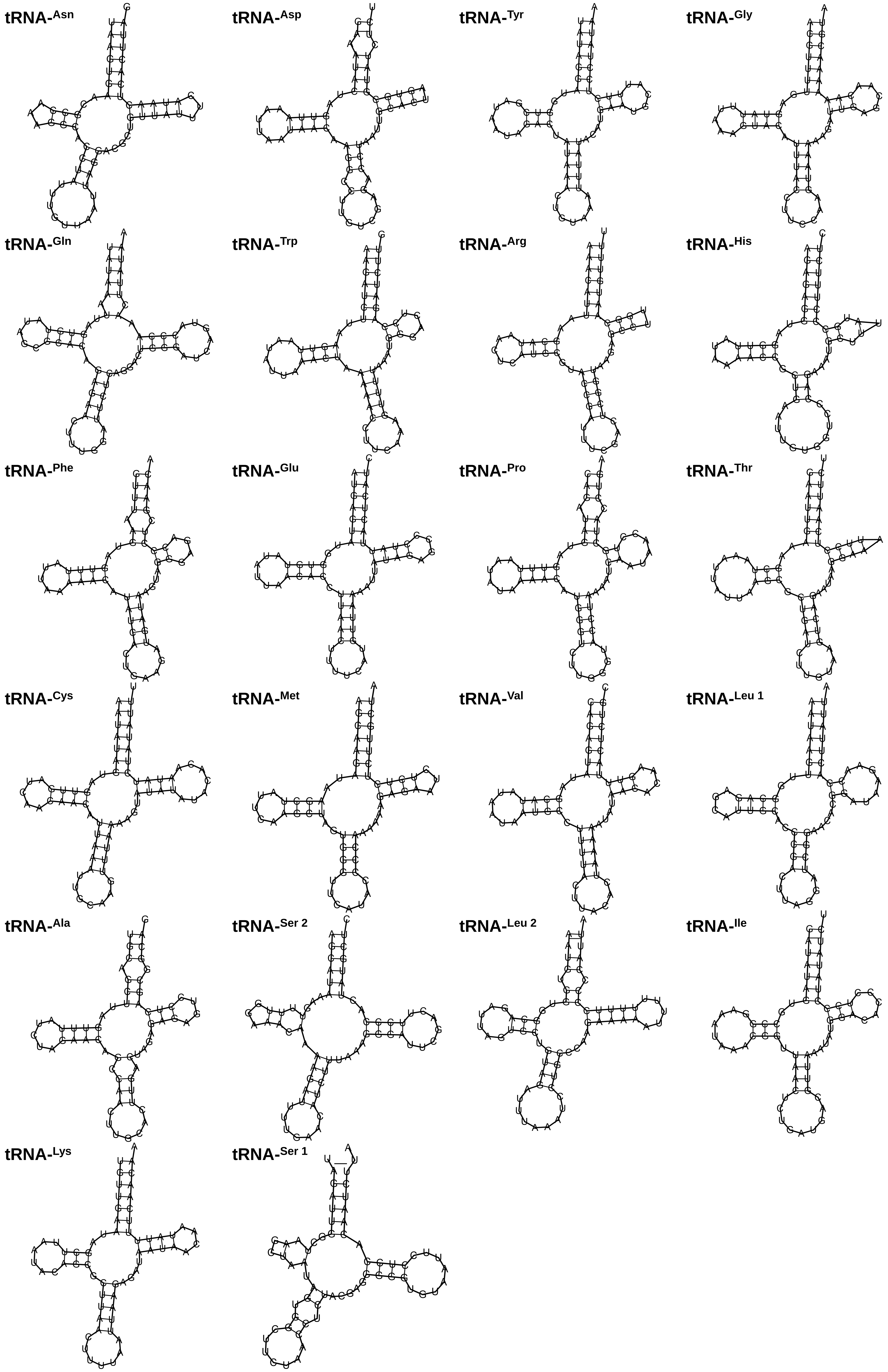
tRNA-Asn
tRNA-Asp
tRNA-Tyr
tRNA-Gly
tRNA-Gln
tRNA-Trp
tRNA-Arg
tRNA-His
tRNA-Phe
tRNA-Glu
tRNA-Pro
tRNA-Thr
tRNA-Cys
tRNA-Met
tRNA-Val
tRNA-Leu 1
tRNA-Ala
tRNA-Ser 2
tRNA-Leu 2
tRNA-Ile
tRNA-Lys
tRNA-Ser 1
